# Phenotypic and Genetic Studies of the Viral Lineage Associated with the Recent Yellow Fever Outbreak in Brazil

**DOI:** 10.3390/v14081818

**Published:** 2022-08-19

**Authors:** Nathália Dias Furtado, Mariela Martínez Gómez, Iasmim Silva de Mello, Déberli Ruiz Fernandes, Myrna Cristina Bonaldo

**Affiliations:** Laboratory of Molecular Biology of Flaviviruses, Oswaldo Cruz Institute, Fiocruz, Rio de Janeiro 21040-900, Brazil

**Keywords:** yellow fever virus outbreak, southeastern Brazil, amino acid polymorphisms, NS3, NS5, virulence

## Abstract

Yellow fever virus (YFV) caused an outbreak in the Brazilian Southeast from 2016 to 2019, of the most significant magnitude since the 1900s. An investigation of the circulating virus revealed that most of the genomes detected in this period carried nine unique amino acid polymorphisms, with eight located in the non-structural proteins NS3 and NS5, which are pivotal for viral replication. To elucidate the effect of these amino acid changes on viral infection, we constructed viruses carrying amino acid alterations in NS3 and NS5, performed infection in different cells, and assessed their neurovirulence in BALB/c mice and infected AG129 mice. We observed that the residues that compose the YFV 2016–2019 molecular signature in the NS5 protein might have been related to an attenuated phenotype, and that the alterations in the NS3 protein only slightly affected viral infection in AG129 mice, increasing to a low extent the mortality rate of these animals. These results contributed to unveiling the role of specific naturally occurring amino acid changes in the circulating strain of YFV in Brazil.

## 1. Introduction

Yellow fever (YF) is an acute febrile disease that has caused hundreds of thousands of deaths worldwide, especially in Africa since the 16th century, and in the Americas in the 18th and 19th centuries [1]. Starting in 1900, studies on the transmission mechanism of YF identified the arthropod vector. Until 1920, campaigns for mosquito control promoted YF eradication in the United States and in several countries of Central and South America, including some regions of Brazil [2]. After developing an effective vaccine against YF, vaccination and vector control eliminated the disease in several continents, restricting the circulation of the yellow fever virus (YFV) to the tropical areas of Africa and South America [1]. Nevertheless, since the 2000s, there has been a reemergence of YF in areas associated with low or no activity of YFV in the endemic countries, and Brazil recently experienced the most critical outbreak in the last 80 years in the southeastern region [3,4]. From 2014 to 2019, YF has been spreading in the country southwards, with the most expressive event in 2017 and 2018. During this period, 2257 confirmed human cases of YF and 1428 epizootics were reported in the southeastern region [5]. From 2019 until 2021, outbreaks of lower magnitude have been detected in South Brazil, with a reemergence in the Central-West, with case reports in Goiás state [6,7].

The dispersal of YFV into southeastern Brazil was more significant than the previous outbreaks in the country since the beginning of the last century. Many factors certainly influenced the outbreak proportions, such as ecological changes that promoted a significant increase in mosquito and non-human primates (NHP) densities, their contact with humans, and low vaccine coverage [8]. On the other hand, the increased circulation and geographic expansion of YFV since the 2000s in South and Southeastern Brazil might also be associated with the emergence of viral genetic variants. YFV, like other flaviviruses, presents evolutive constraints, because they must replicate in very different host cells, and escape from mosquito and mammal antiviral responses. Despite that, it is not unprecedented that an arbovirus evolved to originate a variant with higher fitness in mosquito vectors, due to a few mutations [9]. Even a single mutation in the viral genome enabled the dispersal of the Chikungunya virus (CHIKV) into other continents, causing significant outbreaks [10]. Another good example of this phenomenon is the West Nile virus (WNV); after its introduction in North America, it generated variants that were more competent for transmission by native mosquitos [11,12,13]. 

In a previous study, we described the detection of nine non-synonymous mutations in YFV virus strains over the period of 2016–2017 during the outbreak in southeastern Brazil [14]. Observing this molecular signature in most of the circulating YFV genomes raised questions regarding whether these changes might have influenced the function of viral proteins increasing viral fitness [15]. At that time, we explored the possible impact of the polymorphisms inside NS3 and NS5 on protein functions, using prediction via homology modeling and structural analysis. An analysis of the NS3 protein mapped the amino acid variations (E88D and R121K) inside the protease domain and revealed that the substitution E88D engaged a loss of hydrogen bond with the protein backbone, causing a side-chain displacement. In contrast, the mutation R121K was predicted to favor the formation of a hydrogen bond with the co-factor for proteolytic activity, NS2B, which could modulate the protease efficiency [15]. In addition, experimental studies revealed that when amino acid substitutions E88D and R121K co-occur, the protein displays expressively higher stability, which might have positively selected the current amino acid composition of NS3pro [16]. Amino acid changes inside NS5 mapped inside the MTase domain (K101R, V138I, and G173S) and the RdRp domain (N297S, V643A, and N709S), and some of these amino acid alterations might have an impact on enzymatic efficiency due to their locations [15]. Lastly, we recently published a study comparing YFV isolates detected in different Brazilian states, two carrying the YFV 2016–2019 molecular signature, and two with the genomic composition of previously circulating strains, and we could not conclude whether YFV 2016–2019 molecular signature plays a pivotal role in cell infectivity or neurovirulence [17].

To further clarify the phenotypic effect of the NS3 and NS5 polymorphisms on viral infection and neurovirulence, we synthesized a YFV infectious clone (IC_YFV_2017) based on a strain collected in 2017 in southeastern Brazil. We performed site-directed mutagenesis to revert the residues of the 2017 polymorphisms, generating three YF infectious clone-derived viruses: (i) without the molecular signature in the NS3 (YFV_NS3_2010), (ii) without the molecular signature in NS5 (YFV_NS5_2010), and (iii) without the molecular signature in both NS3 and NS5 (YFV_NS3/NS5_2010). The virus directly derived from IC_YFV_2017 was named YFV_2017. We analyzed the infectivity of the IC_YFV_2017-derived viruses in mammals’ and mosquitos’ cells. Additionally, we inferred the neurovirulence of these viruses and their sensitivity to type I interferon (IFN-I) using two mouse models, BALB/c and AG129. Our main findings suggest that the YFV 2016–2019 molecular signature in NS5 reflected an attenuated phenotype in mammals’ cells and mice. In comparison, cellular assays and the neurovirulence test in BALB/c mice indicate that the YFV 2016–2019 molecular signature in NS3 might have no impact on viral fitness, despite the slight increase in virulence observed in AG129 mice.

## 2. Materials and Methods

### 2.1. Synthesis of the YFV Infectious Clone

A full-length YFV infectious clone based on isolate ES-504 (GenBank: KY885000) was obtained, named IC_YFV_2017, adapting the methodology described by de Mello et al. [18]. Briefly, the viral genomic cDNA was cloned in two plasmids; pCC1/1.4 carries the 3’ and 5’ ends, and pCC1/2.3 carries the central part. Next, fragment 2.3 and the entire plasmid pCC1/1.4 were PCR amplified using Phusion DNA Polymerase (Thermo Fisher Scientific, Waltham, MA, USA), digested with EcoRI (Promega, Madison, WI, USA) and KpnI (Promega, Madison, Wisconsin, USA), and ligated with T4 DNA Ligase (New England Biolabs, Ipswich, MA, USA), assembling the complete genomic viral cDNA. Finally, the template cDNA was further amplified via PCR using GXL PRIMEStar DNA Polymerase (TaKaRa Bio INC, San Jose, CA, USA), in vitro transcribed with the mMESSAGE mMACHINE SP6 Transcription kit (Ambion, Waltham, MA, USA), and electroporated into C6/36 cells to regenerate infectious viral particles.

### 2.2. Insertion of Genetic Markers

Four IC_YFV_2017 clone-derived viruses were obtained, one named YFV_2017, directly obtained from IC_YFV_2017, and three were obtained after site-directed mutagenesis: i) without the molecular signature in the NS3, named as YFV_NS3_2010; (ii) without the molecular signature in NS5, named as YFV_NS5_2010; and (iii) without the molecular signature in both NS3 and NS5, named as YFV_NS3/NS5_2010. The choice of the inserted nucleotides was based on the Venezuelan YFV strain 10A collected in 2010 (GenBank: KM388816) [19]. Clone-derived viruses were obtained via the site-directed mutagenesis of pCC1/2.3 for YFV_NS3_2010 and pCC1/1.4 for YFV_NS5_2010, and both plasmids for YFV_NS3/NS5_2010, using QuikChange II XL Site-Directed Mutagenesis Kit (Agilent, Santa Clara, CA, USA). The oligonucleotide primers used to insert the mutations engaging the amino acid changes D1572E, K1605K, R2607K, I2644V, S2679G, S2803N, A3149V, and S3215N are listed in Table S1.

### 2.3. Viral Stocks

The viral stocks of YFV_2017, YFV_NS3_2010, YFV_NS5_2010, and YFV_NS3/NS5_2010 were obtained from the C6/36 cell infection, previously seeded at 80,000 cells/cm² in T-75 flasks, with 3 mL of cell supernatant harvested after transfection. After 5 days of incubation at 28 °C, the supernatant was collected, centrifuged at 4 °C, 700× *g* for 10 min, filtered with a 0.22 µm syringe filter, and stored at −80 °C. The viral recovery and stock obtention were confirmed via RT-PCR using SuperScript IV and Phusion DNA Polymerase (Thermo Fisher Scientific, Waltham, MA, USA), and sequenced using primer sets and the previously described methodology [14].

### 2.4. Plaque Phenotype Assay

The plaque sizes of the four YF viruses were determined after infection of Vero cells, as previously described [17]. In summary, Vero cells seeded at 40,000 cells/cm² were infected with 10 PFU, 20 PFU, and 40 PFU of each virus. After infection, cells were overlaid with 0.5% agarose prepared in Earle’s 199 medium supplemented with 5% fetal bovine serum (FBS; Cutilab, Campinas, São Paulo, Brazil), 0.25% sodium bicarbonate (Sigma-Aldrich, Darmstadt, Germany), and 40 mg/mL gentamicin (Gibco, Waltham, MA, USA), and further incubated at 37 °C and 5% CO_2_ for 7 days. The cells were fixed in 10.0% formaldehyde and stained with 0.4% crystal violet. Images of the plates were analyzed using ImageJ software version 1.8.0 (National Institutes of Health, Bethesda, AR, USA) to measure the plaque areas. The results were plotted and statistically analyzed using Prism software, version 8 (GraphPad Software, San Diego, CA, USA). Statistical tests employed were Kruskal–Wallis with Dunn’s multiple comparison test.

### 2.5. Viral Growth Kinetics in Different Cell Lines

Vero cells were cultivated in Earle’s 199 medium supplemented with 5% FBS, 0.25% sodium bicarbonate, and 40 mg/mL gentamicin at a density of 40,000 cells/cm² in T-25 cell culture flasks. HepG2 were cultivated in DMEM medium (Gibco, Waltham, MA, USA) supplemented with 10% FBS, 1% (MEM) non-essential amino acids (NEAA; Gibco, Waltham, MA, USA), 1 mM sodium pyruvate (Gibco, Waltham, MA, USA), and 100 U/mL penicillin-streptomycin (Gibco, Waltham, MA, USA), also in T-25 culture flasks. These two cell lines were maintained in an incubator at 37 °C with a wet atmosphere and 5% CO_2_. C6/36 cells were grown in Leibovitz’s L-15 medium supplemented with 5% FBS, 10% tryptose broth (Gibco, Waltham, MA, USA), and 40 mg/mL gentamicin. Aag2 cells were cultivated in Schneider’s Insect medium (Gibco, USA), supplemented with 10% FBS and 100 U/mL penicillin–streptomycin. Both cells were seeded at a density of 80,000 cells/cm² in T-25 flasks, and were maintained in an incubator at 28 °C. All cells were seeded approximately 24 h before infection with YFV_2017, YFV_NS3_2010, YFV_NS5_2010, and YFV_NS3/NS5_2010 at MOI 0.02. The culture medium was discarded and the inocula were prepared in adequate medium with a final volume of 0.5 mL were added to the cell monolayers for viral adsorption. The cells were incubated in the appropriate conditions for 1 h with gentle agitation every 15 min. The viral suspensions were discarded, and 10 mL of cell medium were added to the culture flasks. For 5 days, the cell supernatants were collected daily for viral quantification via plaque assay. This experiment was performed in 3 to 4 replicates for better statistical validation. The viral titers were plotted using Prism version 8 (GraphPad Software, San Diego, CA, USA), and one way ANOVA was used to statistically analyze these data with Dunnett’s multiple comparisons test.

### 2.6. Viral Infection in the Presence of Type I Interferon

Vero cells were grown in 24-well plates at a density of 50,000 cells/cm², the day before infection. First, the cells were treated with human interferon (IFN) alpha (α; PBL Assay Science, Piscataway, NJ, USA) and beta (β; R&D Systems, Mineapolis, MI, USA) at concentrations of 10 UI/mL, 50 UI/mL, 100 UI/mL, and 1000 UI/mL for 6 h before infection. Next, cell supernatants were removed, and 100 µL of viral suspensions were added at MOI of 0.5. After 1 h incubation, the viral inocula were discarded, and 0.3 mL of supplemented Earle’s 199 medium containing the four different IFN concentrations were added to the cells. After 48 h of incubation, the cell supernatants were collected for viral titration via plaque assay. 

The viral titers under treatment with IFN-α and IFN-β were normalized with the values obtained from non-treated and infected cells. This experiment was performed in three independent assays for statistical relevance. Data were analyzed in Prism version 8 (GraphPad Software, San Diego, CA, USA). IC_50_ values were calculated from the nonlinear regression function provided by the software ([Inhibitor] vs. normalized response—Variable slope). IC_50_ values of each replicate were analyzed using a one-way ANOVA with Dunnett’s multiple comparisons test. 

### 2.7. Animal Experimentation

All animal experiments were carried out in strict accordance with the Guide of the National Council for Control of Animal Experimentation (CONCEA). The protocols employed were approved by the Committee on the Ethics of Animal Experimentation of Oswaldo Cruz Institute (CEUA-IOC; Permit: L-034/2019). 

#### 2.7.1. BALB/c Mice

The neurovirulence tests were performed in the BALB/c mouse model. These mice were obtained from CEMIB (Centro Multidisciplinar para Investigação Biológica na Área da Ciência em Animais de Laboratório), of the State University of Campinas, São Paulo (UNICAMP). Three groups of 5 young-adult mice (6 weeks old) were inoculated intracerebrally with 10³ PFU each of YFV, or with the diluent medium, in a final inoculum volume of 30 µL in Earle’s 199 medium supplemented with 25 mM HEPES (Gibco, Waltham, MA, USA) and 0.025% sodium bicarbonate. The inoculation procedure was conducted under anesthesia with a ketamine/xylazine cocktail at 100 mg/kg and 10 mg/kg, respectively, administered intraperitoneally. After injection, mice were monitored daily for weight loss and clinical signs of disease until the humane endpoint or the monitoring period (21 days). The humane endpoint was determined as previously described [17]. Euthanasia was performed with intraperitoneal administration of 3 times the standard dose of ketamine/xylazine cocktail, followed by cervical dislocation. 

#### 2.7.2. AG129 Mice

The virulence experiments were performed with α/β and γ interferon receptor knocked-out mice, AG129. These animals were produced and provided by Instituto de Ciência e Tecnologia em Biomodelos (ICTB) of Oswaldo Cruz Foundation (FIOCRUZ) in Rio de Janeiro, Brazil. The animals were bred and maintained under specific pathogen-free conditions. Two groups of 4-to-5 10-weeks-old mice were inoculated subcutaneously with 60 µL of viral suspension with a 2 × 10^4^ PFU dose or diluent medium (30 µL in each footpad). These animals were monitored daily until the humane endpoint or the end of the 16-day monitoring period. On days 2, 4, 6, and 8, we withdrew blood from the submandibular vein of 3 animals in each group. At the euthanasia of these animals, their blood was collected via heart puncture. The collected blood was used for RNA extraction using the QIAmp Viral RNA Mini Kit (Qiagen, Hilden, Germany) and qRT-PCR to determine viral loads in the specific time points. As previously described, viral detection and quantification via qRT-PCR were performed [20]. The average survival time (AST), percentage of mortality, clinical scores, and percent of body weight were calculated and analyzed in Prism version 8 (GraphPad Software, San Diego, CA, USA). Statistical analysis of Kaplan–Meier survival curves was performed using a log-rank test (Mantel–Cox).

## 3. Results

### 3.1. Influence of the YFV 2016–2019 Molecular Signature on Infectivity in Cell Cultures

To further investigate the role of the amino acid variations observed in the NS3 and NS5 proteins detected in Brazil in 2017 [14,15], we constructed synthetic YFV via reverse genetics and performed site-directed mutagenesis, as described in the Material and Methods section. Table 1 summarizes the amino acid differences between the YF viruses derived from IC_YFV_2017 infectious clone obtained in this work.

**Figure 1:**
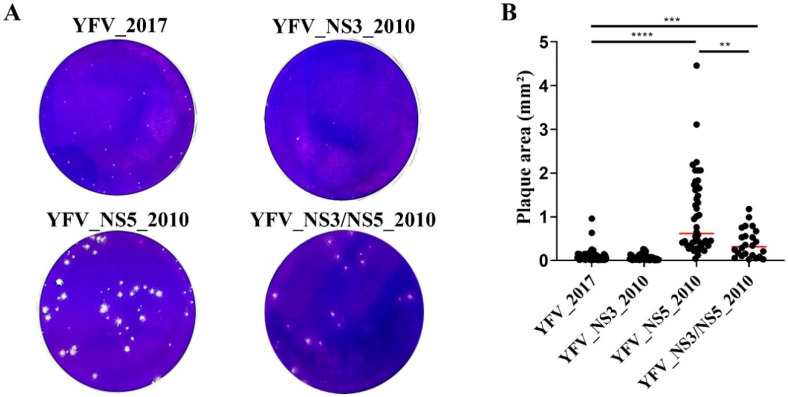
Plaque phenotype of yellow fever clone-derived viruses in Vero cells. (**A**) Images of the analyzed plaque areas for each virus. (**B**) Graphical representation of the measured plaque areas. The red bars represent the median of each sample. The plaque areas were measured using ImageJ software, and the statistical analyses were performed in Prism, version 8(GraphPad Software, San Diego, CA, USA). The data set was analyzed using Kruskal–Wallis’s test, and we compared the YFV_2017 with the other three YFVs using Dunn’s multiple comparisons test. ** represents *p* < 0.01, *** represents *p* < 0.001, and **** represents *p* < 0.0001.

The first phenotypic feature we established was the plaque size formed by each YF clone-derived virus in Vero cells (Figure 1). YFV_2017 displayed a tiny plaque phenotype in Vero cells, similar to those detected with YFV_NS3_2010 (*p* = 0.144). YFV_NS5_2010 showed a larger and more heterogeneous plaque morphology in comparison with YFV_2017 (*p* < 0.0001), while YFV_NS3/NS5_2010, displayed an intermediate plaque size (*p* = 0.0004).

**Figure 2:**
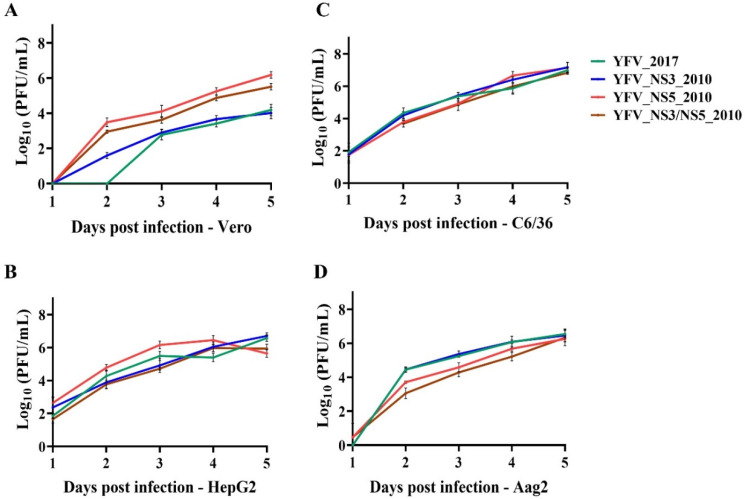
Replication of the yellow fever clone-derived viruses in different cell types. Growth curves were obtained in Vero (**A**), HepG2 (**B**), C6/36 (**C**), and Aag2 (**D**) cells at MOI 0.02, and aliquots of the supernatants were harvested daily for 5 days to quantify plaque-forming units (PFU) at each time point. Viral titers were transformed in log_10_ and plotted using Prism, version 8(GraphPad Software, San Diego, CA, USA). Statistical analyses are available in Figure S1.

We next investigated the infectivity of these viruses in mammalian (Vero and HepG2) and mosquito (C6/36 and Aag2) cell cultures employing an MOI of 0.02, quantifying the viral yield daily until 5 days post-infection. Over time, we observed significantly higher viral titers in Vero cells infected with YFV_NS5_2010 or YFV_NS3/NS5_2010 viruses (Figure 2A and Figure S1). In HepG2 cells, there were smoother differences between the clone-derived viruses, yet YFV_NS5_2010 displayed higher titers than the other three synthetic YF viruses (Figure 2B) until the fourth day post-infection. 

In the mosquitos’ cell line C6/36, derived from Aedes albopictus larvae and very susceptible to flaviviruses’ infection, the viral growth profiles were similar (Figure 2C and Figure S1), with only a few significant differences detected on the second day post-infection, and with YFV_NS5_2010 surpassing the other variants at the fourth day post-infection. In a less susceptible mosquito cell line, the Aag2 cells from Aedes aegypti larvae, the differences were more marked than in C6/36 cells, with YFV_2017 and YFV_NS3_2010 displaying significantly higher titers than the viruses carrying the molecular NS5 2010 amino acid variants, the YFV_NS5_2010 and YFV_NS3/NS5_2010 viruses, from the second to the fourth day post-infection. All the viruses reached viral growth peaks after the 4th day post-infection (Figure 2D and Figure S1).

In summary, we observed that the viruses carrying the YFV 2010-amino acid markers in NS5 tend to better proliferate in the two mammal cells employed in this work. In contrast, those carrying the YFV 2017-amino acid markers in NS5 tend to present higher viral yields in mosquito cells.

### 3.2. Interference of the Molecular Signature on the Innate Immune Response Mediated by Type I Interferon

**Figure 3:**
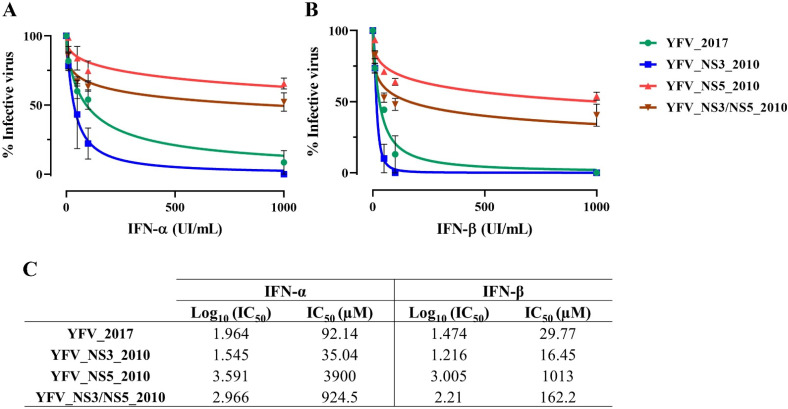
Replication of the yellow fever clone-derived viruses in the presence of type I interferon. Vero cells were treated with 1000, 100, 50, and 10 UI/mL of IFN-α (**A**) and IFN-β (**B**), and infected with each YFV variant at MOI 0.5 for 48 h. The cell supernatants were harvested, and the concentration of viral particles was determined for each condition. Each plot represents the percentage value normalized using the supernatants of infected cells not treated with IFN and fitted in a nonlinear regression for IC_50_ calculation. (**C**) Table with the IC_50_ values for each virus under treatment with either IFN-α or IFN-β. These data were analyzed using Prism, version 8(GraphPad Software, San Diego, California, USA.

We investigated whether the polymorphisms in NS3 and NS5 affect the antiviral response mediated by IFN-I. Vero cells were treated with four concentrations of interferon α or β (IFN-α and IFN-β) and were infected with the four YF clone-derived viruses at MOI 0.5. At 48 h post-infection, we quantified infective viruses in the cell supernatant (Figure 3). The IC_50_ values of each experiment replicate were plotted and compared, to establish statistical differences (Figure S2). 

The viruses carrying the NS5 2010 variations, YFV_NS5_2010 and YFV_NS3/NS5_2010, displayed a lower sensitivity to both types of IFN-I. There is a tendency for YFV_NS5_2010 (IFN-α IC_50_ = 3900 µM; IFN-β IC_50_ = 1013 µM) to be more resistant to IFN-I than YFV_NS3/NS5_2010 (IFN-α IC_50_ = 924.5 µM; IFN-β IC_50_ = 162.2 µM), but the differences were not statistically significant. YFV_NS3_2010 showed the highest sensitivity to IFN-I (IFN-α IC_50_ = 35.0 µM; IFN-β IC_50_ = 16.5 µM), but this was not significantly different from YFV_2017 (IFN-α IC_50_ = 92.1 µM; IFN-β IC_50_ = 29.8 µM) (Figure 3 and Figure S2).

### 3.3. Effects of the Molecular Signature Polymorphisms upon Mouse Inoculation

#### 3.3.1. BALB/c Mice

**Figure 4:**
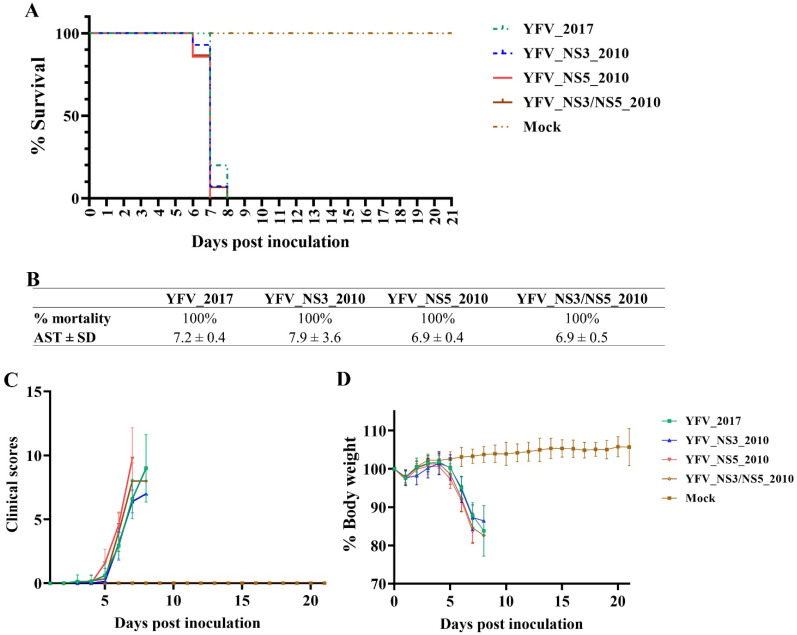
Neurovirulence in BALB/c mice induced by the yellow fever clone-derived viruses. (**A**) Kaplan–Meier survival curves of each virus. (**B**) Table summarizing the survival curve results (AST: Average Survival Time; SD: Standard Deviation). (**C**) Mean of clinical scores and (**D**) mean of percentage body weight presented by each group of infected mice. All analyses were performed in Prism, version 8(GraphPad Software, San Diego, CA, USA. The Kaplan–Meier survival curves were statistically compared using the Log-rank (Mantel–Cox) test.

The intracerebral inoculation of YFV is highly lethal in mice. Even the attenuated vaccine 17D YFV induces a 100% mortality rate in Swiss and BALB/c mice [21,22]. To determine if the molecular markers of YFV 2016–2019 could affect the neurovirulence, we intracerebrally inoculated 6-week-old BALB/c mice with 10³ PFU of each of the YF clone-derived viruses. We measured the body weight, clinical signs of disease, and deaths daily for 21 days. All viruses induced 100% mortality within the first 8 days after injection. According to the clinical scores, all of the infected animal groups presented a similar disease intensity and weight loss of almost 20% (Figure 4). In fact, there was no statistical difference between YFV_2017 and YFV_NS3_2010 (*p* = 0.197) or YFV_NS3/NS5_2010 (*p* = 0.112), according to the Log-rank test (Mantel-Cox). Only YFV_NS5_2010 showed a slight difference from YFV_2017, *p* = 0.028. In conclusion, there are no substantial differences in intracerebral inoculation with the four clone-derived viruses.

#### 3.3.2. AG129 Mice

Next, we investigated the differences in the infection pattern of the YF viruses derived from the infectious clone in another mouse model, the highly susceptible AG129 mice, depleted for the receptors of IFN-α/β and IFN-γ [23]. A total of nine animals per virus received a dose of 2 × 10^4^ PFU subcutaneously and were followed up for 16 days. 

**Figure 5:**
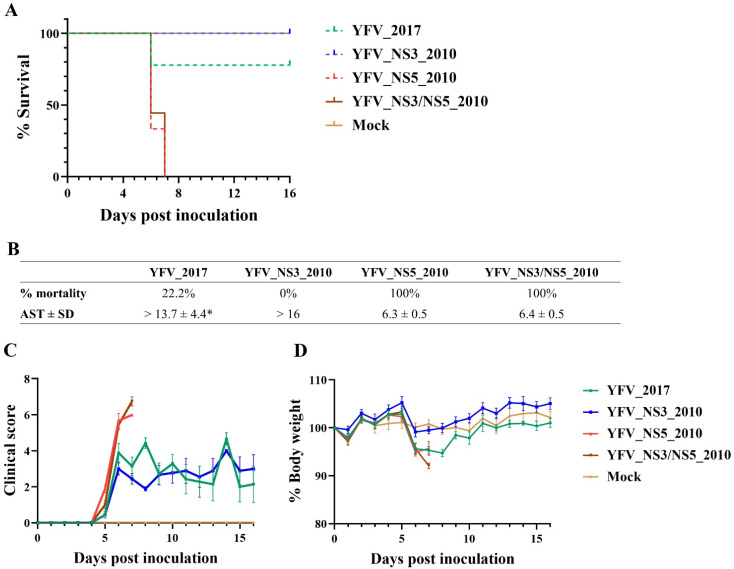
Infection in AG129 mice with yellow fever clone-derived viruses. (**A**) Kaplan–Meier survival curves of each virus. (**B**) Table summarizing the survival curves results (AST: Average Survival Time; SD: Standard Deviation). (**C**) Mean of clinical scores and (**D**) mean of percentage body weight presented by each group of infected mice. All analyses were performed in Prism, version 8(GraphPad Software, San Diego, California, USA. * *p* < 0.1.

Contrary to the first animal evaluation, the YF clone-derived viruses displayed substantial differences in virulence in AG129 mice. The infection with YFV_2017 provoked only a lethality of 22.2% on the sixth day post-infection. The remaining 77.8% of infected mice survived until the end of the follow-up period (Figure 5A,B). On the other hand, the replacement in this genetic background with the YFV 2010 markers in the NS3 caused no lethality in the animals upon infection with YFV_NS3_2010 (Figure 5A,B). Conversely, the YF clone-derived viruses carrying the amino acid polymorphisms of YFV 2010 in the NS5 either present in YFV_NS5_2010 or YFV_NS3/NS5_2010 raised the lethality to 100%, with AST values of 6.3 ± 0.5 and 6.4 ± 0.5 days, respectively (Figure 5A,B). 

The viral infection in all animals was manifested through significantly swollen footpads (at the inoculation sites) and hunched postures, hampering the animals’ mobility, as well as ruffled fur and a modest body weight loss (less than 10%) (Figure 5C,D). Over time, the clinical scores of YFV_2017 and YFV_NS3_2010 were mainly due to swollen footpads and slightly hunched postures, not ruffled fur. Of these two YFVs, only YFV_2017-infected AG129 lost body weight between days 5 and 9 post-inoculation, whereas the YFV_NS3_2010 inoculated animals maintained their body weight to a comparable extent compared to the Mock infected mice (Figure 5D). It is noteworthy that YFV_NS3_2010 infected mice presented more severe swelling at their footpads between the sixth and the ninth days post-infection and recovered almost entirely until the end of the monitoring period.

**Figure 6:**
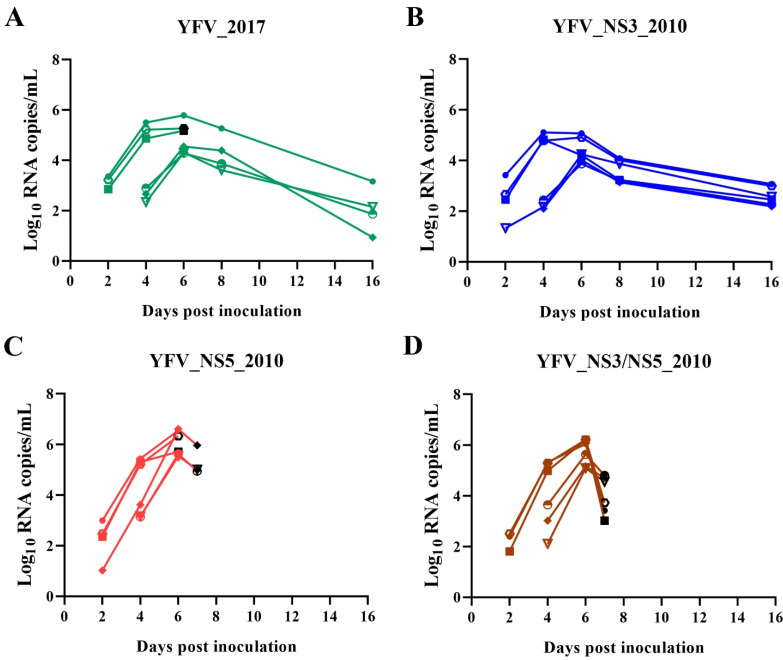
Viremia of infected AG129 over time, determined via qRT-PCR. The concentrations of RNA copies by mL of blood were transformed in log10 and plotted for each time point post-inoculation of animals infected with YFV_2017 (**A**), YFV_NS3_2010 (**B**), YFV_NS5_2010 (**C**), and YFV_NS3/NS5_2010 (**D**). The black symbols represent the mice that reached the humane endpoint and were euthanized before the sixteenth-day post-inoculation.

Further, we measured viremia on days 2, 4, 6, and 8, and at the moment of euthanasia (Figure 6 and Figure S3). The viral loads in the blood of infected AG129 mice reached a peak on the sixth-day post-inoculation. We observed that the viremia peaks of YFV_2017 and YFV_NS3_2010 are significantly lower than the peaks of YFV_NS5_2010 and YFV_NS3/NS5_2010 (Figure S3). 

We observed that the AG129 mice controlled the infection by YFV_NS3_2010 with a peak of viral loads between the fourth and the sixth-day post-inoculation (~10^4^ RNA copies/mL), maintaining a restrained viral load of approximately 500 RNA copies/mL of YFV in the blood at the end of the 16-day monitoring period (Table S2). A very similar pattern of viremia was observed in the surviving mice infected with YFV_2017. In contrast, mice inoculated with YFV_NS5_2010 and YFV_NS3/NS5_2010 succumbed before the eighth-day post-inoculation and presented an increasing viral load in blood until the humane endpoint determined the euthanasia. 

The viremia of all the animals that reached the humane endpoint before the monitoring period was around 10^4^ to 10^6^ RNA copies/mL of blood. Interestingly, mice euthanized on the sixth-day post-inoculation with YFV_2017 displayed a similar average viral load to those inoculated with YFV_NS5_2010, euthanized one day later, on the seventh-day post-inoculation (1.69 × 10^5^ and 3.74 × 10^5^, respectively). Moreover, the animals inoculated with YFV_NS3/NS5_2010 and euthanized on the seventh day exhibited around 10 times fewer RNA copies/mL of blood than the animals euthanized on the same day that were inoculated with YFV_NS5_2010. The highest viral loads were attained by the animals inoculated with YFV_NS5_2010, euthanized on the sixth day, with an average viremia of 2.11 × 10^6^ RNA copies/mL blood (Table S2).

## 4. Discussion

The last YFV outbreak in the country was explosive, and raised questions regarding which factors could be involved, such as adaptative mutations. In the history of arboviruses, it has already happened that a few mutations in the viral genome have led to host adaptation, causing alterations involved in virus dispersion [10,11,12,13]. In previous studies, we observed nine amino acid changes related to the 2016–2019 Brazilian outbreak, and analyzed its possible impacts on virus replicative fitness, using wild-type strains [14,15,17,24,25]. In the present study, we further explore the impacts of NS3pro and NS5, which are previously described amino acid polymorphisms present in YFV strains from the 2016–2019 outbreak, in the context of viral fitness in cells and murine infection models, using an infectious clone approach. 

For this purpose, we first analyzed the plaque phenotype in Vero cells and the replication kinetics of clone-derived viruses in mammalian and mosquito cell cultures. Regarding the NS3 amino acid markers, no impact on the plaque phenotype was observed when Vero cells were infected with YFV_NS3_2010, compared with the YFV_2017 infection. In addition, YFV_NS3_2010 displayed similar growth rates to the YFV_2017 in all cell lines used in this study, and slight differences were observed in sensitivity to IFN-I, where YFV_NS3_2010 tends to exhibit lower IC_50_ values for IFN-α and β. In summary, the NS3 amino acid polymorphisms do not significantly modulate the infection in mammal cells. 

Conversely, the molecular signature in the NS5 2010 protein had more marked effects on mammal viral infectivity, exhibiting higher proliferation rates and larger plaque phenotype in mammal cells. Furthermore, the more highly significant ability to infect mammal cells correlates with more extensive resistance to the inhibition of viral growth in Vero cells under treatment with IFN-I. In general, infection with YFV_NS3/NS5_2010 virus in cellular models displayed an intermediate phenotype compared to YFV_NS5_2010 and YFV_2017, suggesting that NS3-2010 variants somehow modulate the NS5-2010 effect in the mammal infection capacity. Nevertheless, we observed the opposite in YFV carrying NS5 2010 amino acid variations in mosquito cells, in which these viruses displayed a less proliferative ability. However, it is essential to mention that such results should be clarified by using infection in *Aedes*, *Haemagogus*, and *Sabethes* mosquitos, rather than cell lines. Altogether, infectivity results in cells indicate that the 2016–2019 molecular signature might impact the susceptibility of hosts’ and vectors’ cells, promoting an attenuated phenotype in mammals’ cells. 

To better characterize the effect of the YFV 2016–2019 molecular signature on viral infectivity, we assessed their neurovirulence in BALB/c mice. The YFV is highly neurovirulent and lethal after intracerebral injection in adult mice, including the attenuated vaccine strains [22]. This test has been well established to characterize and distinguish YFV strains [26]. Therefore, we decided to investigate whether the genetic differences in Brazil’s recently established YFV circulating could modulate the YFV neurovirulent phenotype. For this, we inoculated intracerebrally BALB/c mice with each of the clone-derived viruses. All viruses cause lethal infection in BALB/c mice; we observed no significant differences in the average survival times. Hence, we concluded that the clone-derived viruses could be further studied regarding infectivity in a different mouse model. 

AG129 mice have been shown to be an effective animal model for studying viremia, disease pathogenesis, and mortality in flaviviruses [23,27,28,29,30,31]. The AG129 mice lack IFN−α/β and γ receptors, but they elicit B-cell and T-cell responses to infection [32]. Following mammal-cell model studies, the infection of AG129 mice with viruses carrying the NS5 2010 amino acid residues was more lethal, differing from infection with those viruses bearing NS5 2016–2019 molecular markers. Animals infected with YFV_NS5_2010 and YFV_NS3/NS5_2010 viruses exhibited 100% mortality rates (AST around the sixth day p.i) and 10–100 fold higher viremia peaks. Inoculation with the YFV_2017 virus caused a reduced mortality rate (22.2%), indicating a decrease in mouse virulence.

On the other hand, the more pronounced effect in attenuation was reached by the YFV_NS3_2010 administration, which did not provoke any animal death. Together with the data obtained in mammal-cell infection, the NS5-2010 amino acid markers contribute to a more virulent phenotype. Our data from mammal cells suggest that the NS5-2010 molecular signature may better antagonize the type I IFN signaling pathway. As previously described elsewhere [33,34,35], the modulation of the IFN-I signaling pathway by the binding of YFV NS5 to STAT-2 promotes the modulation of the antiviral response. On the other hand, in AG129 mouse infection, deficient of IFN−α/β and γ receptors, perhaps other receptors such as RIG-I and MDA5 could be critical for initiating the IFN response to an infection via the recognition of intracellular viral RNA. NS5 2010 could display a more efficient ability in modulating this antiviral response [35]. 

In summary, we conclude that the presence of the molecular signature in the YFV genome played an essential role in viral fitness in invertebrate and vertebrate hosts mainly associated with NS5 protein. Interestingly, a genomic study involving the South American and West African lineages revealed that the primary amino acid changes relevant to the adaptive diversification of YFV generally cluster in different structural regions of NS5, suggesting an essential role in YFV dissemination [36]. In previous work, using YFV isolates from the Brazilian 21st century outbreaks, the amino acid factors associated with virulence were mainly mapped to non-structural proteins, most of them at NS5. Here, we use infectious clone technology to better determine the impacts of some of these amino acid alterations, especially regarding the NS3 and NS5 2016–2019 signatures. In this genetically controlled landscape, it was possible to draw more precise conclusions on the effect of the YFV 2016–2019 molecular signature. 

## Figures and Tables

**Table 1:** Amino acid changes between the yellow fever clone-derived viruses used in the current study.

Synthetic YFV	NS3		NS5					
	Protease		MTase			RdRp		
	88	121	101	138	173	297	643	709
YFV_2017	D	K	R	I	S	S	A	S
YFV_NS3_2010	E	R	R	I	S	S	A	S
YFV_NS5_2010	D	K	K	V	G	N	V	N
YFV_NS3/NS5_2010	E	R	K	V	G	N	V	N

The residues of the previously circulating strain of YFV (Venezuelan strain 10A) are highlighted in blue.

## Data Availability

Not applicable.

## Supplementary Materials

The following supporting information can be downloaded at: https://www.mdpi.com/article/10.3390/v14081818/s1, Figure S1: Statistical analysis of the viral replication kinetics in Vero (A), HepG2 (B), C6/36 (C), and Aag2 (D); Figure S2: Statistical analysis of IC_50_ values generated after viral infection under treatment with IFN-I; Table S1: Oligonucleotide sequences for site-directed mutagenesis in NS3 and NS5 proteins; Table S2: Follow-up of viral RNA copies/mL in blood samples from infected AG129 mice; Figure S3: Statistical analysis of viremia values of viral RNA copies/mL in blood samples from infected AG129 mice.
