# Supplementary material for: Phenotypic and Genetic Studies of the Viral Lineage Associated with the Recent Yellow Fever Outbreak in Brazil
Source: Viruses. 2022 Aug 19;14(8):1818. doi: 10.3390/v14081818 (PMC9412561; doi:10.3390/v14081818)
Supplement: Supplementary file 1 [file viruses-14-01818-s001.zip › viruses-1832641-supplementary.pdf]

Supplementary Material

Phenotypic and genetic studies of the viral lineage associated with the recent yellow fever outbreak in Brazil

Authors: Nathália Dias Furtado[1], Mariela Martínez Gómez[1], Iasmim Silva de Mello[1], Déberli Ruiz Fernandes[1], Myrna Cristina Bonaldo[1]*

[1] Laboratório de Biologia Molecular de Flavivírus, Instituto Oswaldo Cruz-Fiocruz, Rio de Janeiro, Brazil

* Correspondence author: mbonaldo@ioc.fiocruz.br

Table S1. Oligonucleotide sequences for the site-directed mutagenesis in NS3 and NS5 proteins.

Forward Primer		Reverse Primer	
**ID**	**Sequence (5' → 3')**	**ID**	**Sequence (5' → 3')**
NS3_D88E(+)	GGCGGCTCATGGAAGTTGGAGGGTAGA TGGGAT	NS3_D88E(-)	ATCCCATCTACCCTCCAACTTCCATGAG CCGCC
NS3_K121R(+)	CCAAGCCTATTCAAGGTTAGGAATGGA GGAGAAATTGGG	NS3_K121R(-)	CCCAATTTCTCCTCCATTCCTAACCTTG AATAGGCTTGG
NS5_R101K(+)	GAGAAGTGAGTGGGGTCAAGGGATTCA CCCTT	NS5_R101K(-)	AAGGGTGAATCCCTTGACCCCACTCACT TCTC
NS5_I138V(+)	CCATCGCCTTGAGCCGGTAAAGTGTGAT ACCCT	NS5_I138V(-)	AGGGTATCACACTTTACCGGCTCAAGG CGATGG
NS5_S173G(+)	CTGTTGAGAAATGGTTGGGCTGTGGTGT TGAAAGC	NS5_S173G(-)	GCTTTCAACACCACAGCCCAACCATTTC TCAACAG
NS5_S297N(+)	CACCGCCACTTGGTTCCATGATAATGAC AACCCTTA	NS5_S297N(-)	TAAGGGTTGTCATTATCATGGAACCAA GTGGCGGTG
NS5_A634V(+)	GGACTGTGACGACACGGTTTTGACCAAG CTTGAAG	NS5_A634V(-)	CTTCAAGCTTGGTCAAAACCGTGTCGTC ACAGTCC
NS5_S709N(+)	AAAAGGATGGGATGACTGGGAGAATGT GCCCTTTTG	NS5_S709N(-)	CAAAAGGGCACATTCTCCCAGTCATCC CATCCTTTT

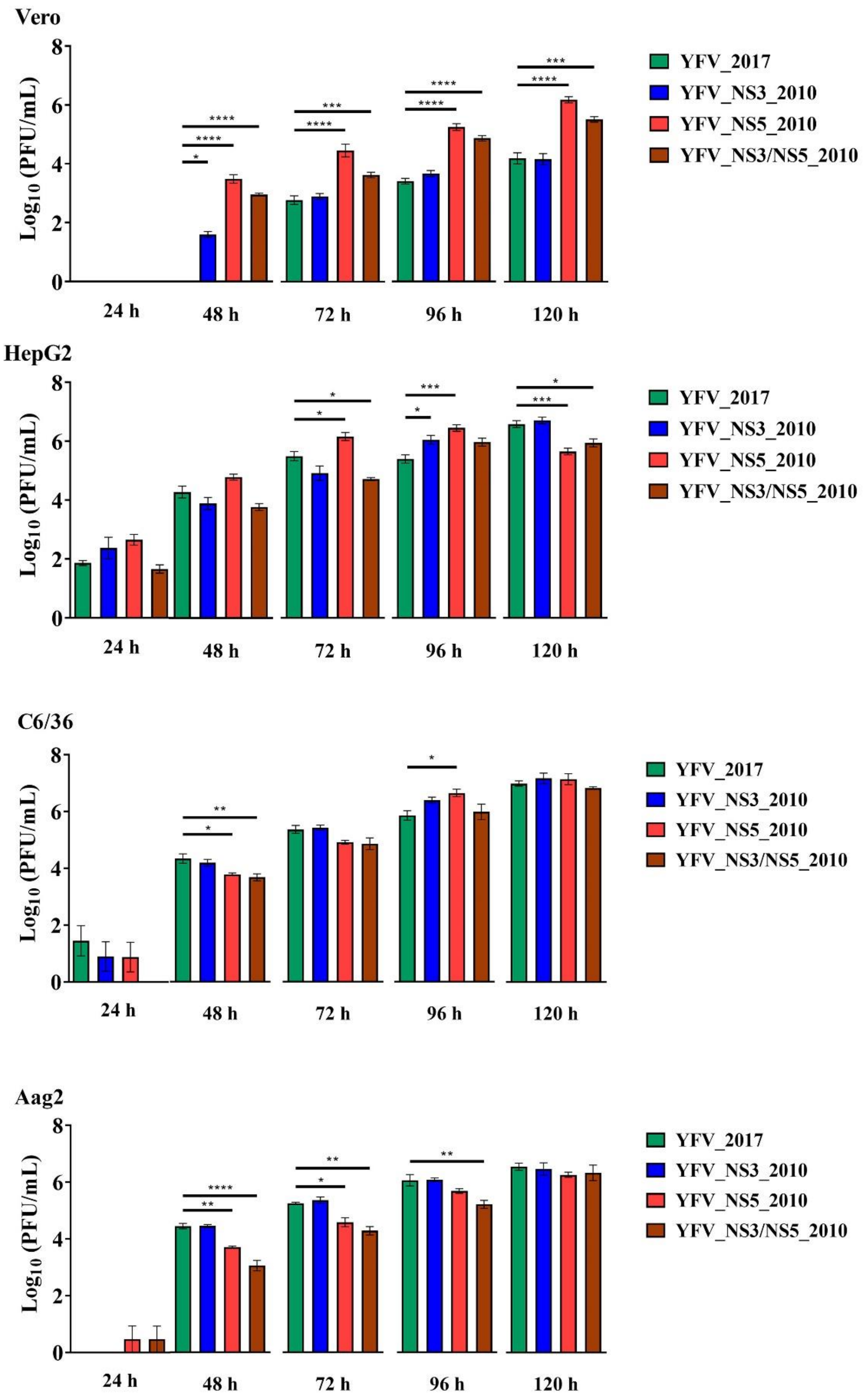

Figure S1. Statistical analysis of the viral replication kinetics in Vero (A), HepG2 (B), C6/36 (C) and Aag2 (D). Viral titers average of each YFV isolate were compared using One-way ANOVA with Dunnett's multiple comparisons test: * represents $p \leq 0.05$, ** represents $p \leq 0.01$, *** represents $p \leq 0.001$ and **** represents $p \leq 0.0001$.

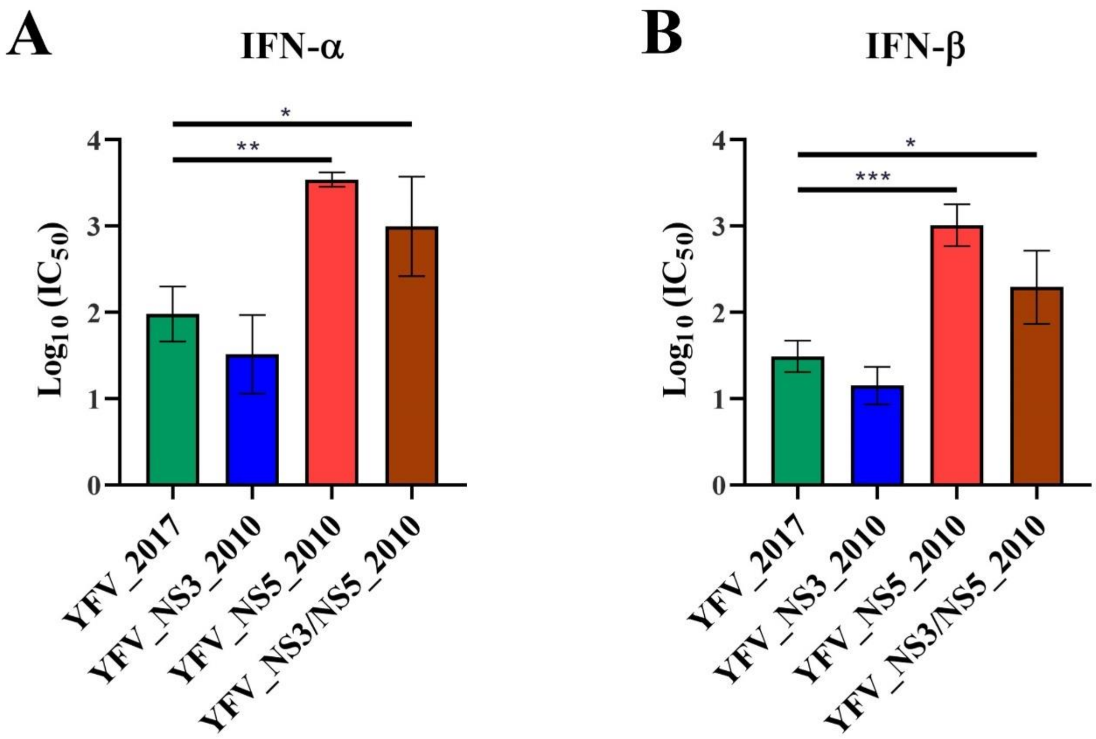

Figure S2. Statistical analysis of IC_{50} values generated after viral infection under treatment with IFN-I. The IC_{50} values of each replicate were plotted in a column table and analyzed for statistical differences. The test applied was One-way ANOVA with Dunnett's multiple comparisons test: * represents $P \leq 0.05$, ** represents $P \leq 0.01$, *** represents $P \leq 0.001$ and **** represents $P \leq 0.0001$.

Table S2. Follow-up of viral RNA copies/mL in blood samples from infected AG129 mice

Inoculated virus	Mouse	Survival time (days)	Viremia in RNA copies/mL by day post-inoculation (d.p.i.)					
			2	4	6	7	8	16
YFV 2017	1	16	0.00	7.96×10^2	1.91×10^4	n.d.	7.35×10^3	7.28×10^1
	2	16	0.00	2.20×10^2	2.05×10^4	n.d.	4.03×10^3	1.42×10^2
	3	16	0.00	4.53×10^2	3.53×10^4	n.d.	2.48×10^4	8.62×10^0
	4	6	7.11×10^2	7.42×10^4	1.53×10^5	●	●	●
	5	6	1.64×10^3	1.65×10^5	1.86×10^5	●	●	●
	6	16	2.30×10^3	3.13×10^5	**$6.15 \times 10^{5*}$**	n.d.	1.86×10^5	1.45×10^3
YFV NS3 2010	1	16	0.00	2.74×10^2	7.35×10^3	n.d.	1.63×10^3	1.85×10^2
	2	16	2.15×10^1	1.49×10^2	1.73×10^4	n.d.	7.33×10^3	3.72×10^2
	3	16	0.00	1.25×10^2	1.06×10^4	n.d.	1.39×10^3	1.54×10^2
	4	16	2.91×10^2	6.56×10^4	1.61×10^4	n.d.	1.68×10^3	2.90×10^2
	5	16	4.53×10^2	6.04×10^4	8.09×10^4	n.d.	1.02×10^4	1.01×10^3
	6	16	2.65×10^3	**$1.28 \times 10^{5*}$**	1.18×10^5	n.d.	1.21×10^4	1.18×10^3
YFV NS5 2010	1	7	0.00	1.37×10^3	4.32×10^5	8.94×10^4	●	●
	2	7	0.00	1.46×10^3	3.51×10^5	1.03×10^5	●	●
	3	7	1.04×10^1	4.19×10^3	**$4.06 \times 10^{6*}$**	9.31×10^5	●	●
	4	6	2.29×10^2	2.02×10^5	5.26×10^5	●	●	●
	5	6	2.93×10^2	1.64×10^5	2.20×10^6	●	●	●
	6	6	1.01×10^3	2.78×10^5	3.59×10^6	●	●	●
YFV NS3/NS5 2010	1	7	0.00	4.44×10^3	4.40×10^5	6.38×10^4	●	●
	2	7	0.00	1.29×10^2	1.25×10^5	3.63×10^4	●	●
	3	7	0.00	1.05×10^3	1.48×10^5	5.04×10^4	●	●
	4	7	6.42×10^1	9.77×10^4	1.64×10^6	1.05×10^3	●	●
	5	7	3.24×10^2	1.99×10^5	**$1.66 \times 10^{6*}$**	5.37×10^3	●	●
	6	7	2.49×10^2	2.15×10^5	1.13×10^6	2.64×10^3	●	●

n.d.= non-determined viremia; ● = post-euthanasia period; * viremia peak

Average viremia at euthanasia for each YFV infected mice (RNA copies/mL):

Virus	Day post-inoculation	Viremia (RNA copies/mL)
**YFV_2017**	16	$4{,}19 \times 10^2$
	6	$1{,}69 \times 10^5$
**YFV_NS3_2010**	16	$5{,}32 \times 10^2$
**YFV_NS5_2010**	6	$2{,}11 \times 10^6$
	7	$3{,}74 \times 10^5$
**YFV_NS3/NS5_2010**	7	$2{,}66 \times 10^4$

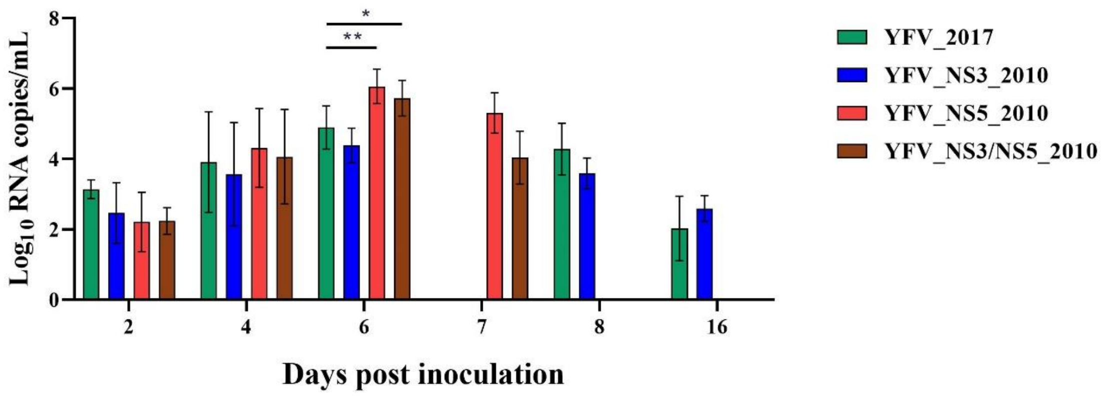

Figure S3. Statistical analysis of viremia values of viral RNA copies/mL in blood samples from infected AG129 mice. The $\log_{10}$ values of viral loads in the blood of AG129 mice were compared by One-way ANOVA with Dunnett's multiple comparisons test: * represents $P \leq 0.05$, ** represents $P \leq 0.01$.
